# Developmental frameworks, what have you done for me lately?

**DOI:** 10.1017/S0954579425101016

**Published:** 2025-12-18

**Authors:** Isabella C. Stallworthy, Meriah L. DeJoseph, Marion I. van den Heuvel, Daniel Berry, Willem E. Frankenhuis

**Affiliations:** 1 Department of Bioengineering, University of Pennsylvania, Philadelphia, PA, USA; 2 Graduate School of Education, Stanford University, Stanford, CA, USA; 3 Tranzo Scientific Center for Care and Wellbeing, Tilburg University, Tilburg, Netherlands; 4 Institute of Child Development, University of Minnesota, Twin Cities, MN, USA; 5 Department of Evolutionary and Population Biology, Institute for Biodiversity and Ecosystem Dynamics, University of Amsterdam, Amsterdam, the Netherlands; 6 Max Planck Institute for the Study of Crime, Security and Law, Freiburg, Germany

**Keywords:** Developmental psychology, frameworks, philosophy of science, metascience

## Abstract

Frameworks are widespread in developmental psychology. They provide general ideas about what to study in human development: which concepts to focus on (e.g., systems, timescales), which processes to test (e.g., micro–macro, bidirectional), and which methods to use (e.g., interview, dynamical equations). However, despite their prominence, there exists very little consensus or guidance on how to use frameworks in research. As such, they have an obscure role, influencing our research questions, methods, and theory, but often in ways we cannot articulate for ourselves, let alone for others. This Views paper presents our perspective on how different frameworks can inform the assumptions, targets, goals, context, timing, and methods of a research project. As an illustrative example, we use Bronfenbrenner’s bioecological framework to inform research investigating how parent–child relationships shape the development of executive self-regulation. We also show how different frameworks relevant to developmental psychopathology can inform a research project in distinct ways. Thus, this Views paper provides a practical guide for developmental researchers to more explicitly use and benefit from frameworks in their research.

## Introduction

*“… our curriculum has glossed over a crucial step: The student, now a junior researcher, has learned how to operate the hypothesis-testing machinery but not how to feed it with meaningful input.”* (Scheel et al., 2021, p. 744)

For decades, psychological scientists have championed the value of developmental frameworks, the idea being that scientific practice benefits from big-picture conceptual guidance. At the same time, researchers at all career stages appear to be grappling with the quandaries described in the quote above, such as how to use the many frameworks that exist in our field to conduct principled research. This is a perennial challenge for the field, spanning generations of researchers. Reflect back on any undergraduate or graduate course in developmental psychology and you will recall some mention of “developmental systems” or “Bronfenbrenner’s bioecological model,” and likely several other “guiding frameworks.” They are often trainees’ first introduction to the big-picture topics in developmental psychology and can spark an initial interest in research. First learning about these frameworks ignites excitement and intrigue but soon gives way to uncertainty about where to go from there. Beyond their inspiring ideas, what real purpose do frameworks serve? How should we use them to strengthen our research? The goal of this Views paper is to provide our perspective on how frameworks are currently used in developmental psychopathology, and psychology at large, and how they could better guide research in the future.

Frameworks are ubiquitous in developmental research and teaching (Badcock, 2012; Bjorklund, 2018; Ploeger et al., 2008; Schilbach et al., 2013; Witherington, 2007; Witherington et al., 2018). A Google Scholar search at the time of the writing of this Views paper reveals over 1.3 million hits for journal articles that contain the terms ‘framework’ and ‘child development.’ Frameworks offer general ideas about human development – what to study and how – and are thought to constitute the background to scientific research (Bjorklund, 2018; Lerner et al., 2015; Overton, 2007; Witherington et al., 2018). Some argue that all researchers subscribe to one framework or another, even if only implicitly (Guest & Martin, 2021). As a result, frameworks are either left out of research or invoked in an opaque manner (see below). These practices lead to unclear links between frameworks and research, hindering transparency and the pursuit of a cumulative science. We view these shortcomings as an opportunity to propose a guide for using frameworks to improve developmental research. This Views paper offers tools that, from our perspective, help to clarify how to use frameworks in developmental research.

### What are frameworks, exactly?

**Figure 1.**
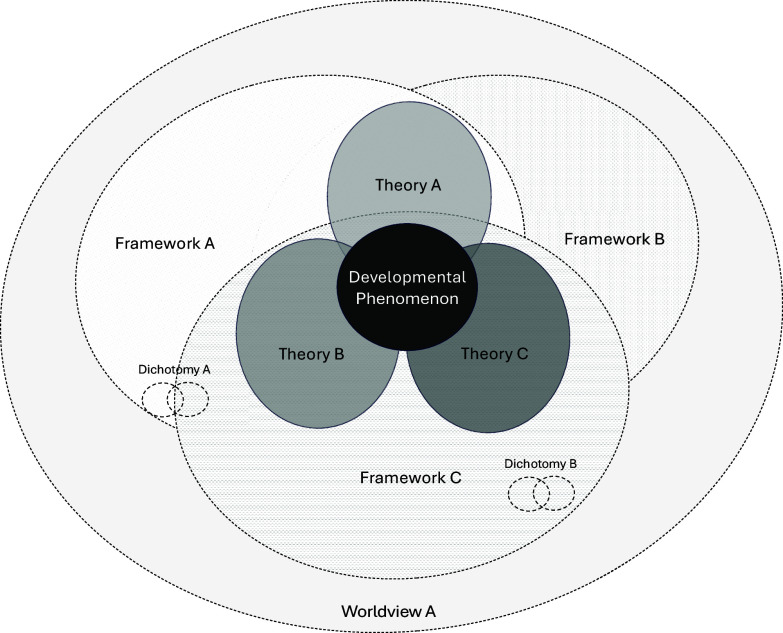
Frameworks within developmental research. *Note.* Depiction of the relations between scientific worldviews, frameworks, theories, and dichotomies in the study of developmental phenomena. Worldviews, or paradigms (e.g., Cartesian-split-mechanistic; Overton, 2013; Reese & Overton, 1970; Witherington, 2007), constitute the broadest category. Within a worldview, a framework is a set of broad overarching ideas, principles, concepts, and assumptions, about which things to study and potentially how to study them. Within worldviews and frameworks, theories are more specific sets of testable scientific propositions that describe, explain, predict, and or control a target phenomenon (Eronen & Bringmann, 2021; Guest & Martin, 2021; Van Rooij & Baggio, 2021). Dichotomies (e.g., nature vs nurture) characterize developmental ideas that exist within and across these categories.

Frameworks are challenging to define because they take different forms and have different labels^1^, and are often conflated with theories (Muthukrishna & Henrich, 2019). Despite their differences, the term “theory” is frequently used in the literature to refer to frameworks (Muthukrishna & Henrich, 2019; Witherington et al., 2018). Here, we define a framework as a set of broad overarching ideas, principles, concepts, and assumptions, about which things to study and potentially how to study them (Bjorklund, 2018; Thelen et al., 1991; Witherington et al., 2018). A framework may be thought of as a conceptualization of the world and a set of rules for studying it. Frameworks describe and prescribe what is acceptable and desirable for theory and research (Overton, 2007). We can think of a framework as a spotlight on a set of general ideas for thinking about, and potentially studying, developmental phenomena. By contrast, a theory is a more specific set of testable propositions that describe, explain, predict, and or control a target phenomenon (Bringmann et al., 2022; Eronen & Bringmann, 2021; Guest & Martin, 2021; Muthukrishna & Henrich, 2019). Thus, strictly speaking, frameworks are not theories (Figure 1). And although there is now ample guidance on the value of theories and their connections to research (Borsboom, 2013; Borsboom et al., 2021; Frankenhuis et al., 2013a; Guest, 2024; Guest & Martin, 2021; Fried 2020; Scheel et al., 2021; Smaldino et al., 2015), there exists no equivalent guidance for frameworks.

We can consider the dynamic systems **(DS)** framework (Thelen, 2005), which often gets mislabeled as DS *theory*. DS originated as an area of mathematics to describe the behavior of complex, dynamical systems or collections of interacting parts, often using differential equations (Close et al., 2001). Over time, DS has also grown into several general frameworks, including one for studying human development (Smith & Thelen, 2003; Thelen, 2005). The DS framework applied to human development views development as a dynamical system, maintaining that humans have properties in common with other dynamical, complex systems, such as nonlinear change (Table 1). Like most frameworks, DS contains general ideas, not testable claims or specific predictions about any particular developmental phenomenon (Guest & Martin, 2021). Notably, the DS framework has been used to create more specific theories, for example, about how humans develop knowledge from everyday actions (Thelen & Smith, 1996) or how the physical world contributes to locomotion in infants (Thelen et al., 1991). Thus, frameworks can encompass different theories within their spotlights, and a given theory can be informed by multiple different frameworks (see Figure 1).

**Table 1.** Summaries of four example frameworks in developmental research

Framework	Scope	Developmental Domains	Summary
Bronfenbrenner’s bioecological approach (BA)	Human development	All	Emphasizes studying human development in its ecological context. There are many interacting, nested environments, ranging from the immediate to their broader historical context, influence human development (Bronfenbrenner, 1977; 1999; Bronfenbrenner & Ceci, 1994; Bronfenbrenner & Morris, 2007).
Dynamic systems (DS)	All complex systems (human to non-living systems)	All	Humans are complex dynamic systems, and their development emerges from interacting nonlinear processes that span multiple systems and timescales. Dynamic systems contain self-organizing, interaction-dominant processes, and emergent properties that cannot be reduced to the sum of their parts (Lewis, 2000; Smith & Thelen, 2003; Thelen, 2005; Thelen & Smith, 1996, 2006; van Geert, 2019; Witherington, 2007).
Life-history theory (LHT)	All organisms including humans	Those related to evolutionary fitness (survival and reproduction)	Focuses on how organisms allocate their limited resources, such as time and energy, across different activities relevant to evolutionary fitness, such as growth rate, somatic repair and maintenance, reproduction, and parenting (Belsky et al., 1991; Ellis & Del Giudice, 2019; Frankenhuis & Nettle, 2020; Galipaud & Kokko, 2020; Nettle & Frankenhuis, 2020; Stearns & Rodrigues, 2020).

*Note.* Summaries of the scope, developmental domains, and main ideas of three frameworks common in developmental psycho(path)logy, used as examples in this Views paper.

Another example is life-history theory (**LHT**), a branch of biology used by evolutionary psychologists to understand how humans allocate their limited resources, such as time and energy, across different activities relevant to evolutionary fitness such as growth, repair and maintenance, reproduction, and parenting (Belsky et al., 1991; Ellis & Del Giudice, 2019; Frankenhuis & Gopnik, 2023). However, strictly speaking, the term “theory” in “life history theory” is a misnomer, because LHT is actually a broad framework for studying allocation trade-offs (de Vries et al., 2023; Frankenhuis & Nettle, 2020; Nettle & Frankenhuis, 2020; Stearns & Rodrigues, 2020). As Stearns and Rodrigues (2020) put it: “Life history theory is an overarching set of ideas, an organizational paradigm, about what questions to ask, what assumptions to make, and what simplifications to accept.” (p. 483). Thus, even in the biological sciences, frameworks are sometimes, confusingly, referred to as theories.

Frameworks differ from one another along several dimensions, such as their *scope* (e.g., humans, all organisms, living and non-living systems), *relevance* to different domains (e.g., emotion, cognition, action), and the *specificity* of ideas (e.g., nonlinear processes, development in context) (see Table 1). For instance, the DS framework considers humans as one of many complex DS that share certain general properties (Mitchell, 2009; Thelen & Smith, 2006; Thurner et al., 2018). DS is not specific to human development, or even to living organisms, but can be tailored to study development (Fogel, 2011; Thelen, 2005; Thelen & Smith, 1996, 2006; Witherington, 2007). LHT also focuses on living organisms; and in particular, how evolutionary selection pressures have shaped developmental mechanisms. Lastly, Bronfenbrenner’s bioecological approach **(BA)** focuses on humans and how to study their development within nested, interacting ecological contexts (Bronfenbrenner, 1977).

The BA, DS, and LHT frameworks complement the prominent developmental psychopathology framework (Cicchetti, 1984; Cicchetti & Toth, 2009; Rutter & Sroufe, 2000), which some, including its founder, Dante Cicchetti, suggest has evolved into an entire discipline (Cicchetti & Toth, 2009; Crnic, 2024; Hyde et al., 2024; Pluess, 2024). Our main points apply broadly to developmental psychopathology and others, including but not limited to: ecological approach (Adolph, 2019; Gibson, 1988; Gibson & Pick, 2000), resilience (Masten, 2001; Masten et al., 2021; Ungar et al., 2013), second-person neuroscience (Schilbach et al., 2013), systems (Von Bertalanffy, 1968; 1972), and developmental systems (Ford & Lerner, 1992) frameworks. The diversity of frameworks in developmental psychology can be exciting but also daunting, as it means there is no one-size-fits-all protocol for how to use them in research. Nevertheless, in what follows, we provide practical guidance from our perspective. (We refer readers interested in a discussion of frameworks within the context of philosophy of science to Overton, 2013 and Reese & Overton, 1970.)

### How developmental researchers currently use frameworks

If research papers mention frameworks at all, they tend to do so ambiguously. Developmental research is often claimed to “draw on,” “follow from,” “be derived from,” “through the lens of,” “based on,” “inspired by,” or “grounded in” some framework. However, the nature of the framework and its links with the research are rarely explicit, leaving it open to multiple interpretations (Frankenhuis et al., 2023; Guest, 2024; Guest & Martin, 2021). The ambiguous use of frameworks occurs in part because their ideas are underspecified. For instance, there are many ways to interpret and operationalize each idea in the service of a given research project. The BA framework discusses the idea of interconnected, nested environments ranging from the individual to their broader socio-historical context (Bronfenbrenner, 1977). Yet it is not straightforward how this idea should inform research. As Kagan (1978) lamented: “The interactions between biological and environmental forces determine the psychological growth of the organism: What in heaven’s name does that fourteen word sentence mean?” (p. 44). How are “nested environments” defined and how do we decide which ones to measure? What kinds of “interactions” do we refer to with respect to which “environments”? Which causal relations between the environments and the individual should be prioritized, if any?

The ambiguous use of frameworks also manifests in researchers using them inconsistently within a single paper. For instance, a framework might appear in the methods section (e.g., “we used dynamic systems methods”), but not other parts of the paper, leaving the reader wondering how these parts are connected. Other times, researchers cite a framework in the introduction and discussion sections (e.g., citing DS to introduce cognitive dynamics in development), while ignoring or even violating its principles in the methods (e.g., obscuring dynamics by aggregating behavioral responses) (Richters, 1997). Some studies are explicit about deducing specific claims from a framework (e.g., Beebe et al., 2016; Chow et al., 2005; Perone et al., 2021; Vermeent et al., 2024), but this level of precision is an exception.

Using frameworks ambiguously threatens our ability to conduct rigorous research in pursuit of robust knowledge of adaptive and maladaptive development. There may even be incentives to be “strategically ambiguous” about how one uses frameworks to guide research (Frankenhuis et al., 2023). Ambiguity about concepts and their relations creates wiggle room in interpretation; for instance, about how hypotheses and findings relate to a framework or how different hypotheses within a framework relate to each other. Perhaps unintentionally, researchers can exploit this wiggle room to make the evidence for hypotheses seem stronger than it really is, to weld loosely related hypotheses into polished narratives, or to exaggerate the empirical success of a particular framework.

There are numerous potential problems of the ambiguous use of frameworks for rigorous scientific practice, which extend to every facet of the research process. Unclear links between frameworks and the introduction leave ambiguities about the motivations for the research questions. Unclear links between frameworks and research designs and measures leave ambiguities about the study’s epistemological commitments, the ontological status of its core constructs, and their roles in measured phenomena. And unclear links between framework-derived hypotheses and the study’s analyses leave ambiguities about the validity of the intended inference – the degree to which the analyses are actually testing the phenomenon they are intended to test. Such ambiguities make it difficult or impossible to see how the study extends current knowledge or links to other work purportedly drawing from the same framework, slowing cumulative science. However, rather than abandoning frameworks altogether, we see an opportunity – in the spirit of Winston Churchill’s famous phrase, “Never waste a good crisis” – if used more explicitly and deliberately, frameworks can provide a powerful tool for principled research and cumulative scholarship.

### How can we leverage frameworks explicitly in research?

Frameworks can offer a roadmap for turning big-picture thinking into concrete research choices. Put simply, frameworks help us decide where to “zoom in” and where to “zoom out.” To use a framework explicitly, we need to link its ideas to specific components of a research project (Table 2). We illustrate this process primarily using BA to inform an example research project studying how parent–child relationships shape children’s executive self-regulation, a transdiagnostic dimension critical for a variety of psychopathologies (Beauchaine, 2015; Beauchaine & Cicchetti, 2019).

**Table 2.** Potential components within which frameworks influence developmental research

Component	Definition
*Assumption*	A concept, or relation between concepts, not directly tested in an empirical study but which undergirds more specific and testable claims (e.g., conceptualizations of the mind, development, and knowledge; population- vs individual-level process).
*Research Target*	The high-level question or focal process within a developmental phenomenon that a researcher wishes to explore or explain (e.g., level of analysis, developmental window, unit of analysis) (Lundberg et al., 2021).
*Research Goal*	The goal of the research project which can include description, prediction, classification, and causal or non-causal explanation (e.g., causal concepts, predictive models) (Ross, 2025).
*Context*	Features (e.g., physical, social, environmental, sociopolitical) external to or separate from the explanatory target measured objectively or subjectively, that are important for understanding its functioning (e.g., microsystem, mesosystem, macrosystem, chronosystem) (Bronfenbrenner & Morris, 2007).
*Timing/ Change*	The concepts about timescales and processes of change that are most relevant to understanding a developmental phenomenon (e.g., timescales, nature of change, source of variation) (see Overton, 2007; Thelen & Smith, 2006).
*Methods*	The design, measurement, and analytical tools for answering a research question about an explanatory target (e.g., statistical models, empirical estimand, study design, measures).

*Note.* Example set of core components, or considerations for a developmental research project, and their definitions.

### Assumptions

We might begin by identifying how a framework’s assumptions inform the decision-making in a research project. Assumptions can be about which types of things exist (e.g., neural networks, cognitive modules) or about relations between those things (e.g., nonlinear interactions, mind–body connections). Assumptions are often not tested directly, and are sometimes not testable, yet they shape the questions we ask and how we answer them (Richters, 1997; Thelen et al., 1991; Thelen & Bates, 2003; Witherington et al., 2018). Assumptions constitute the “hard core,” or the foundational backbone of a given framework (Lakatos, 1970). From these assumptions, we may derive more specific theories and hypotheses, and potentially methods, for testing them. Those theories and hypotheses, serve as a “protective belt” around the framework’s core assumptions (Lakatos, 1970): if the data fail to support them, we typically adjust the hypotheses or methods rather than abandoning the framework altogether.

The BA framework’s main assumption is that humans, like other animals, are best studied within the specific environments in which they live. These ecologies can range from proximal, real-time exchanges between the child and caregiver to more distal cultural and historical contexts. BA framework assumes that contexts nested within one another interact to influence child development and mal(adaptation). More specifically, it assumes that each context impacts development, that contexts can influence each other, and the ways in which they influence each other can impact development (which we further explore in the subsequent section). Applying the BA assumption to our example project, we reason that the effect of parent–child relationships on executive self-regulation can only be understood within the specific nested and interacting environments in which they occur. This assumption is similar to those of some other frameworks (e.g., LHT, DS, developmental psychopathology) but contrasts with others (e.g., core knowledge). This kind of reflection in the research process helps make implicit beliefs visible and actionable.

### Context

“Context matters” is an old but ambiguous adage in developmental research. Contexts (also termed “ecologies,” “ecosystems,” or “environments”) refer to features that are external and separate from the unit of analysis. Frameworks can help identify which ones are most important, how they relate to the other constructs in the project, and how they should be analyzed. Famously, the BA framework focuses on the roles of nested contexts in development and takes an expansive view of which contexts are important. However, it raises questions about nesting relative to whom or what? With respect to typical as well as atypical development? In our example study of parent–child relationships in relation to executive self-regulation, the researcher could study contexts nested around the child (e.g., parents, daycare), parent (e.g., work environment, cultural norms about “good parenting”), or parent–child relationship itself (e.g., home, neighborhood).

BA also invites us to consider how contexts and development *interact* with one another to inform adaptive and maladaptive development. But what exactly interacts with what and what is meant by “interacts”? Here, it helps to translate abstract interactions into testable ideas. Using our example, we might consider several non-mutually exclusive ideas: *(i)* We can study how contexts moderate the association between parent–child relationships and executive self-regulation (e.g., how the strength of the association between parent–child relationship quality and children’s executive attention varies with economic hardship). *(ii)* Alternatively, we could test how aspects of the parent–child relationship explain the effects of a context on children’s executive self-regulation (e.g., whether the effects of economic hardship on executive attention are explained by the parent’s autonomy-supportive behaviors). *(iii) Or* we might study how a context directly shapes our core constructs of interest (e.g., whether high multi-modal stimulation in the home exacerbates clinical and sub-clinical challenges with executive self-regulation). Spelling out which kind of interaction you refer to makes “context matters” a researchable claim rather than a shallow slogan.

### Research target

A framework can influence a project’s research target. In developmental psychology, the research target refers to the specific process or question a researcher wants to understand–often a dynamic one over time, and often in relation to a given outcome (Table 3). This is sometimes referred to as the *theoretical estimand*, or the focal object of inquiry for a given project (Lundberg et al., 2021). However, scientists receive little training in how to determine a research target (Alon, 2009).

**Table 3.** Connecting different frameworks to a research project on Parent–child relationships

Framework	Assumptions	Target	Context	Timing/ Change	Goal	Methods
**BA**	Development is driven by nested, interacting contexts.	Parent–child interactions as a function of proximal and distal environments.	Nested, interacting micro-, meso-, macro-, chrono-systems.	Change unfolds across nested levels that vary with development.	Pathways through which contexts influence development.	Multilevel modeling, interaction terms.
**DS**	Development is nonlinear, emergent, and interaction- dominant.	Multilevel, moment-to-moment processes coordinated between parents and children.	Physical and social environments as constraints or co-creators of development.	Phase transitions and changes at multiple timescales.	Cascades, pathways, mathematical description.	Time-series modeling, continuous time models, nonlinear analyses.
**LHT**	Development is shaped by trade-offs between survival and reproduction.	Parental investment strategies and their associations with child behavior.	Evolutionary pressures, resource availability, adaptive responses to environmental inputs.	Longitudinal adaptation, intergenerational transmission of information.	Pathways or prediction.	Evolutionary formal modeling, longitudinal data.

*Note.* The examples provided in this table serve as illustrations and are not exhaustive. Researchers can create a similar table by selecting a developmental topic and mapping how different frameworks inform assumptions, targets, goals, context, timing, and methods – highlighting both similarities and differences to guide decisions about integration between these components of frameworks.

The BA framework positions individual development within the child’s most proximal environments as the primary focus of analysis. The people, objects, and symbols in the child’s immediate microsystem environment constitute the primary “engine of development” (Bronfenbrenner & Morris, 2007, p. 825). That is, children’s ecological niches are unique, and proximal processes within those niches are the strongest drivers of individual development. At the same time, BA also reminds us that broader ecological forces, such as community support or larger policies, can shape typical and atypical development at a population level through diverse proximal processes. Focusing on individual development, we could also draw on the BA ideas of the importance of reciprocal proximal relations and children’s active engagement with their environments. From this, we might opt for the parent–child dyad as the unit of study to capture their reciprocal interactions and associations with executive self-regulation. As we illustrate below, we can also build upon these ideas by integrating more BA ideas as well as ideas from other complementary frameworks (see Table 3). This can help fill gaps in an area in a research project that our main framework does not sufficiently inform.

### Timing/Change

Frameworks can also influence a project’s ideas about the timescales and the types of change that are most relevant. These ideas could include the frequency of measurement (e.g., generational or across years, second-by-second), the nature of change (e.g., incremental, discontinuous), and how variables relate across different timescales (e.g., moment-by-moment parent-interactions shaping attention developing across months). While BA is often discussed in terms of its focus on context, it also highlights that developmental processes unfold across multiple timescales. Rather than privileging any single timescale, BA invites researchers to clarify which timescale is most meaningful for their specific question. Accordingly, BA can be usefully combined with frameworks that focus on a specific timescale within a given context.

For example, we can draw on ideas from DS, which emphasizes the “moment-by-moment” timescales, nested within daily activities, themselves nested within discontinuous shifts in development (Smith & Thelen, 2003; Thelen, 2005; Thelen et al., 1991). When applied to our working example, DS might lead us to revise our target from parent–child interactions to the study of physiological synchrony between parents and children and its relations with moments of executive self-regulation and the way it can break down in the context of psychopathology. Alternatively, LHT offers a different lens by focusing on longer timescales and evolutionary pressures that shape developmental mechanisms. For instance, if variability in parental responsiveness tracks environmental stress (Belsky et al., 1991) and unpredictability, children might use these fluctuations to allocate attention to uncertain contexts (Frankenhuis et al., 2013b). Incorporating this kind of reasoning helps clarify which timescale–and which kind of change–matters most for a given question.

### Research goal

Frameworks can also shape whether the overarching goal of the research project is to provide a causal or non-causal explanation (e.g., mechanism), exploratory or mathematical description (e.g., dynamics), predictive analysis (e.g., forecasting), or classification (e.g., clinical groups) of the developmental phenomenon (Ross, 2025). Being explicit about this goal helps clarify what kind of knowledge the study aims to produce. Developmentalists often seek causal explanations, but frameworks differ in how they conceptualize causation (Ross, 2021). Some common ideas about causation, or causal concepts, include pathways, mechanisms, cascades, circuits, causal cycles, and structuring causes (Ross, 2025). We consider some example causal concepts below.

One popular but seldom defined causal concept in frameworks is the *mechanism* (Ross & Bassett, 2024). Originating in the study of machines, causal mechanisms are identified by “drilling down” to the lower-level features of a system by decomposing, localizing, and separately studying its constitutive parts and their interactions (Ross, 2021). Some frameworks point towards mechanisms, such as BA, which may advocate for investigating the proximal mechanisms by which context shapes executive functioning, for example. From this, researchers might consider parent–child interactions as a key mechanism responsible for how the home environment impacts executive functioning.

Another causal concept, emphasized by LHT, is the *causal pathway* – a sequence of interconnected causal links that track the flow of signal through a system (Ross, 2021). LHT could introduce causal pathways through its focus on developmental plasticity – the capacity of individuals to use early-life experiences to guide later developmental trajectories and outcomes. In our example, applying LHT might mean tracing how environmental unpredictability influences parenting behaviors, which in turn tune children’s attention over time.

Lastly, some frameworks feature the *cascade* causal concept – a common one in developmental psychopathology (Masten & Cicchetti, 2010). A causal cascade often involves multiple causes operating across different levels of analysis that interact within or between levels to amplify an effect, producing a self-sustaining quality or “snowball effect” (Ross, 2021). DS posits that interactions across multiple timescales and levels of analysis can lead to the emergence of new skills. Accordingly, DS might focus on how parent–child physiological and behavioral synchrony unfold at different timescales and interact together to cause qualitatively new forms of executive self-regulation, such as the transition from normative to clinically disabling challenges with inhibitory control. Thinking clearly about the overarching research goal and how the framework connects to that goal, such as the causal ideas aligned with the framework, keeps researchers honest about what kind of knowledge they aim to produce and informs the methods they use to do so.

## Methods

Thus far, the research project components that we wish to link to the framework are often described within the primary article that outlines a given framework, even if loosely so. Intriguingly, rarely do articles about a given framework talk explicitly about the methods or analytic tools researchers should use to test their questions. The methods of a research project can include study design, statistical models and their parameter selection, and measurement instruments. Some (but not all) frameworks can help us with our methodological choices to concretely align our research question with the methods used to test it. Because methods depend heavily on the research project components (e.g., the estimand) discussed above, they are quite reasonably chosen last and the rationale for choosing a given method is not often explicitly stated. Researchers have the opportunity to adopt methods that map closely to a given framework and state clearly how the ideas in a framework influence their methodological choices.

As illustrated in Table 3, DS ideas tend to align with time-series or nonlinear modeling that can capture the continuous, emergent, and cascading nature of developmental change (Hasselman, 2023). DS also tends to favor idiographic methods, which focus first on modeling at the individual level before averaging to the sample. In contrast, BA emphasizes nested ecological contexts that can be captured using multilevel models with interaction terms and the ability to accommodate individuals clustered within nested groups (Teachman & Crowder, 2002). LHT, which is concerned with adaptive processes on developmental timescales in response to environmental conditions, is often tested with longitudinal models of empirical data or evolutionary formal modeling (Frankenhuis & Walasek, 2020).

Still, as noted, the overarching research goal may not be explanation but prediction – particularly in applied work aimed at understanding who benefits from prevention or intervention (Yarkoni & Westfall, 2017). For instance, to reduce executive self-regulation challenges among children in low-income contexts, a researcher might use regression or machine learning to identify key predictors. Even if these tools are not tightly linked to a framework, frameworks can still guide decisions about which variables to include and how to interpret them. For example, BA might prompt inclusion of predictors across ecological levels while LHT might prioritize indicators of harshness or intergenerational transmission of information about environmental conditions. In short, frameworks don’t just motivate our questions; they guide how we test and interpret them.

### Summary & practical takeaways

**Figure 2.**
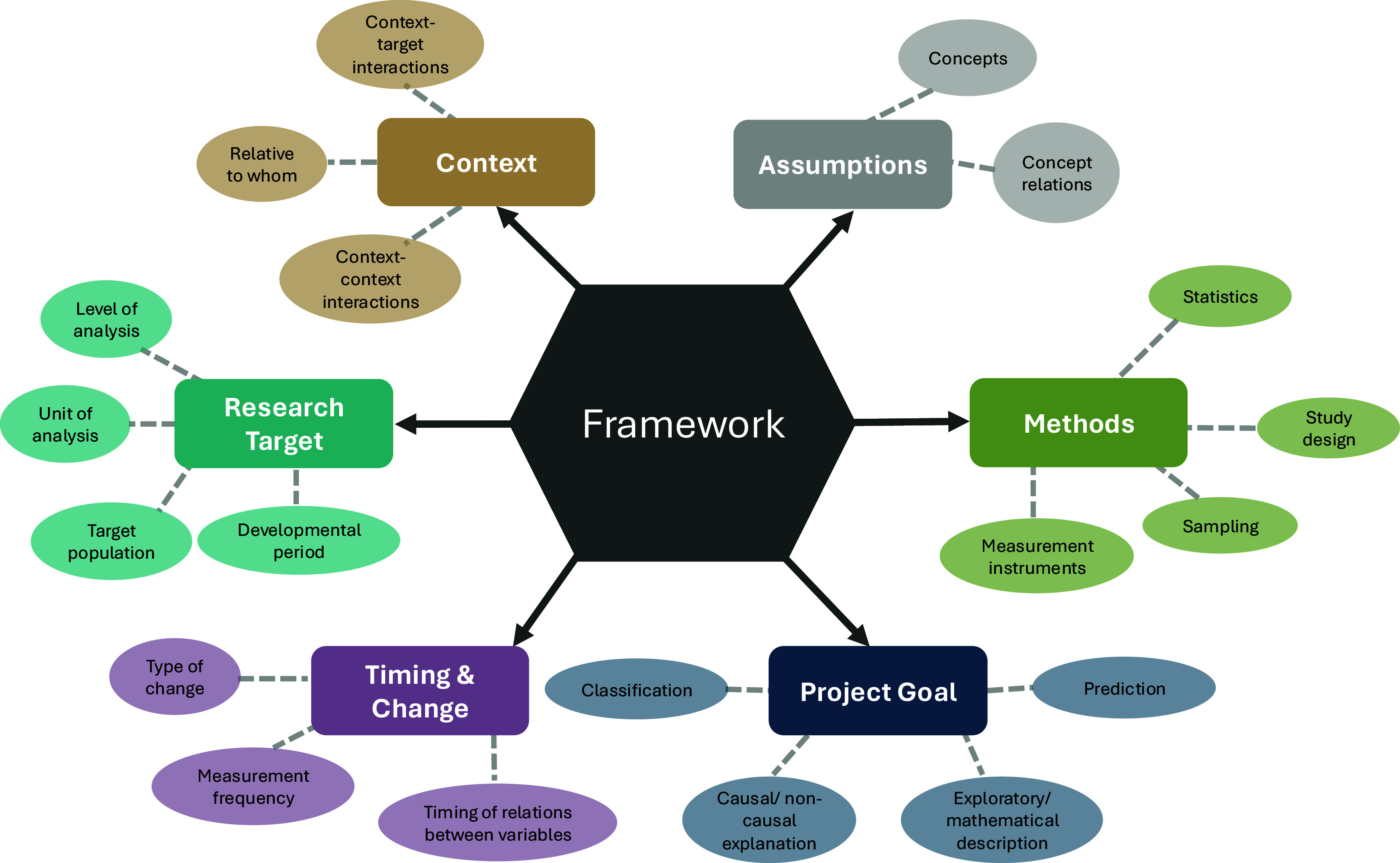
Summary of guidance for using frameworks to inform a developmental research project. *Note.* Frameworks can inform the components of a developmental research project ranging from the ‘hard core’ of the assumptions to specific decision-making in the project’s implementation. Multiple complementary frameworks can be integrated to inform the components of a single research project.

In summary (Figure 2), our goal has been to show not only *where* frameworks can inform developmental research, but also *how* to use them in a more intentional and transparent way. We illustrate how multiple frameworks can shape the assumptions of a research project, the target of study, and the project’s overarching goal. Returning to the core components through which a framework can influence research throughout a project helps make visible the reasoning that guides decisions. This kind of reflection can clarify thinking for those trainees new to the world of developmental frameworks, and it can also help more experienced researchers step out of familiar habits that may have become vague or automatic over time. We urge researchers at all stages of their careers to go the step further of explicitly delineating for themselves and their trainees, mentors, collaborators, funders, readers, and publishers how frameworks inform their research process. *What does being explicit look like in practice?* To assist readers in their own efforts, we have summarized our guidance for each of the six components in a practical exercise (see Supplemental Material). Below we outline several concrete places where researchers can use this exercise to begin to build these habits.

**1. Before starting a project.** Use the guiding questions and exercise to map how a framework’s ideas connect to your research question or help to build and refine it further. This exercise can help identify your assumptions, the key concepts you will focus on, where more clarity is needed, or whether another framework might strengthen the approach.

Example: If using BA, sketch out which ecological levels are relevant for your question and jot down where your current data does or does not capture them. This brief “map” often reveals gaps or assumptions that would otherwise stay implicit.

**2. When designing and preregistering a study.** Clearly name the framework shaping the project (i.e., domains described above) and specify which components of it influence your study design. This supports alignment between conceptual ideas and research choices and makes transparent where decisions reflect theoretical reasoning versus practical constraints.

Example: If a framework emphasizes moment-to-moment change, specify in a preregistration that you selected experience sampling or repeated observations to capture these dynamics. If that design is not feasible, note that the study will only align with part of the framework.

**3. When writing the manuscript:**

a. Introduction: Name the framework(s) early and use it to explain why you are focusing on particular assumptions, constructs, or relations. This helps readers follow the line of reasoning throughout the paper and justify the choice of framework(s).

Example: If drawing on DS, briefly explain why variability and change are central to the phenomenon rather than focusing only on average levels or traits.

b. Methods: Link study design, measures, and analytic choices to the framework components that guided them. If some ideas cannot be directly tested, acknowledge this openly so readers understand the scope of your conclusions.

Example: If a framework emphasizes reciprocity but your data lack measures of a dyadic process, note that you are capturing only one slice of a process that typically unfolds over time and between people.

c. Discussion: Revisit the framework to interpret the findings. Highlight where results support expectations, point to potential refinements, or suggest directions that extend the framework.

Example: If results show that one aspect of the framework was supported but another was not, discuss how this adds nuance to the theoretical landscape and what future work might test next.

Developing these habits strengthens clarity and helps research across the field “speak to each other” more effectively. Making our use of frameworks explicit in how we plan studies, train students, review work, and write manuscripts can help build a shared practice that is easier to learn from and build upon. The supplemental exercise can be further adapted for project planning, lab meetings, preregistration, or manuscript development so that teams can use a common structure while still tailoring the approach to their research.

## Challenges & future directions

We hope this Views paper will spearhead an ongoing conversation in the field about how to conduct and evaluate framework-informed research. We identify the following ongoing challenges that provide valuable opportunities for future work.

### Identifying frameworks

Identifying frameworks and their ideas can be challenging. Sometimes it can be difficult to distinguish between frameworks with similar ideas (Thelen & Bates, 2003), to track changes in a framework over time (Eriksson et al., 2018) or decipher differences between versions of a framework (Witherington, 2007). Further, given the broad use of the term “theory” and related terms (e.g., “model,” “meta-framework”), it can be difficult to distinguish frameworks from theories. What starts off as a theory may grow into a framework, as more general claims are added, which apply to a wider range of phenomena. Our proposal for using frameworks explicitly invites further work identifying the boundaries between theory and framework.

### So many frameworks! deciding when to use them

How do we evaluate the merit of frameworks? Given the more abstract ideas of frameworks, it is difficult to definitively rule out the utility of any given framework for a research topic or developmental research as a whole. In addition to the alignment of the framework with ideas we value, criteria could include the uniqueness of the framework’s ideas and the capacity to bridge across disciplines. In practice, this might mean asking: Does this framework help me see my phenomenon in a new way, guide concrete design choices, or connect insights across fields that might otherwise remain separate? We can also evaluate the implications of a framework’s ideas for real-world translation and policy. This requires asking the critical question about whether the potential conclusions from a particular framework can benefit specific individuals, groups, or dimensions of clinical relevance.

We can also compare the usefulness of different frameworks for a given project. Frameworks may often be similar to one another yet disagree with each other, for instance, encapsulating researchers’ differing ideas about the roles of mental representations (Lerner et al., 2015; Thelen & Bates, 2003; Thelen & Smith, 2006), the body (Gallagher, 2017), and experience (Adolph, 2019) for development. Some frameworks may be most useful for understanding specific domains of development, or for navigating a particular project component, described above. Others may be most useful as loose metaphors or as a pedagogical tool for teaching about development, which offers general ideas but nothing that can directly inform a research project. Alternatively, a framework might be most useful within its particular historical moment (Scarr, 1985), as a fresh counter-perspective to the research zeitgeist at the time, but no longer offers novel guidance for the modern researcher. We can be pluralistic and draw from the affordances of different frameworks. However, we are faced with the additional challenge of determining how different frameworks can (and cannot) go together.

### Cumulative science – Can frameworks help?

The field is rife with calls to go beyond mere fact-finding toward cumulative understanding in developmental research (Curran, 2009; Muthukrishna & Henrich, 2019; Roisman, 2021; Schmidt, 1996). This goal carries both basic and translational science implications. Some suggest that the “ultimate contribution” of a framework is to synthesize theories and empirical results in the service of a coherent “big picture” (Ketelaar & Ellis, 2000; Ploeger et al., 2008). Frameworks, when used explicitly and deliberately, do offer an organizing structure that can bridge existing theories and empirical work. They can provide a systematic way of organizing different theoretical traditions in science, helping researchers survey our growing understanding of human development within and between frameworks (Muthukrishna & Henrich, 2019). This alignment holds potential to enhance the field’s collective contributions.

## Conclusion

Frameworks are everywhere. They can play a role in all aspects of the scientific process–from our earliest introductions to research and the questions we ask, to the assumptions we make and methods we use. Ultimately, they influence how we interpret results and understand a given developmental phenomenon. We hope that this Views paper offers developmental researchers a better idea of what frameworks can do for them, and of how to use frameworks in their research. We hope they see how to build clearer connections between frameworks and a research project, moving away from vague terms like “draw on,” “through the lens of,” or “inspired by” to use frameworks in more explicit, transparent, principled, and less ambiguous ways. While we have not offered up a single, fixed algorithm for applying frameworks to developmental research, we have endeavored to provide a general guide for the “how” of harnessing the affordances and diversity of frameworks in any research program. We do not view this as the ultimate or final guide for using frameworks in research but rather as a pragmatic conversation-starter that fills a much-needed gap. To conclude, we hope this work helps reignite and channel the enthusiasm for developmental frameworks sparked by our training, this time with a practical starting guide for how to infuse them into our research.

## Supporting information

10.1017/S0954579425101016.sm001 Stallworthy et al. supplementary material Stallworthy et al. supplementary material

## Data Availability

There are no data used in this paper.

## Footnotes

1
Frameworks, as we define them, are sometimes also referred to as “theories” (e.g., Rochat, 2023), “metatheories” (Bjorklund, 2018; Ketelaar & Ellis, 2000; Overton, 2007; Witherington, 2007; Witherington et al., 2018), “perspectives” (Witherington, 2007), “models” (Bronfenbrenner & Morris, 2007), “meta-models” (Witherington et al., 2018), or “theoretical frameworks” (Muthukrishna & Henrich, 2019), although notably, others use these same terms to mean other things.

## Supplementary material

The supplementary material for this article can be found at https://doi.org/10.1017/S0954579425101016.

## Pre-registration statement

This study was not pre-registered given that it is a non-empirical paper.
