# Supplementary material for: Developmental frameworks, what have you done for me lately?
Source: Dev Psychopathol. 2025 Dec 18:1–10. Online ahead of print. doi: 10.1017/S0954579425101016 (PMC12825961; doi:10.1017/S0954579425101016)
Supplement: Stallworthy et al. supplementary material [file S0954579425101016sup001.docx]

**Exercise: Leveraging Frameworks for a Developmental Research Project**


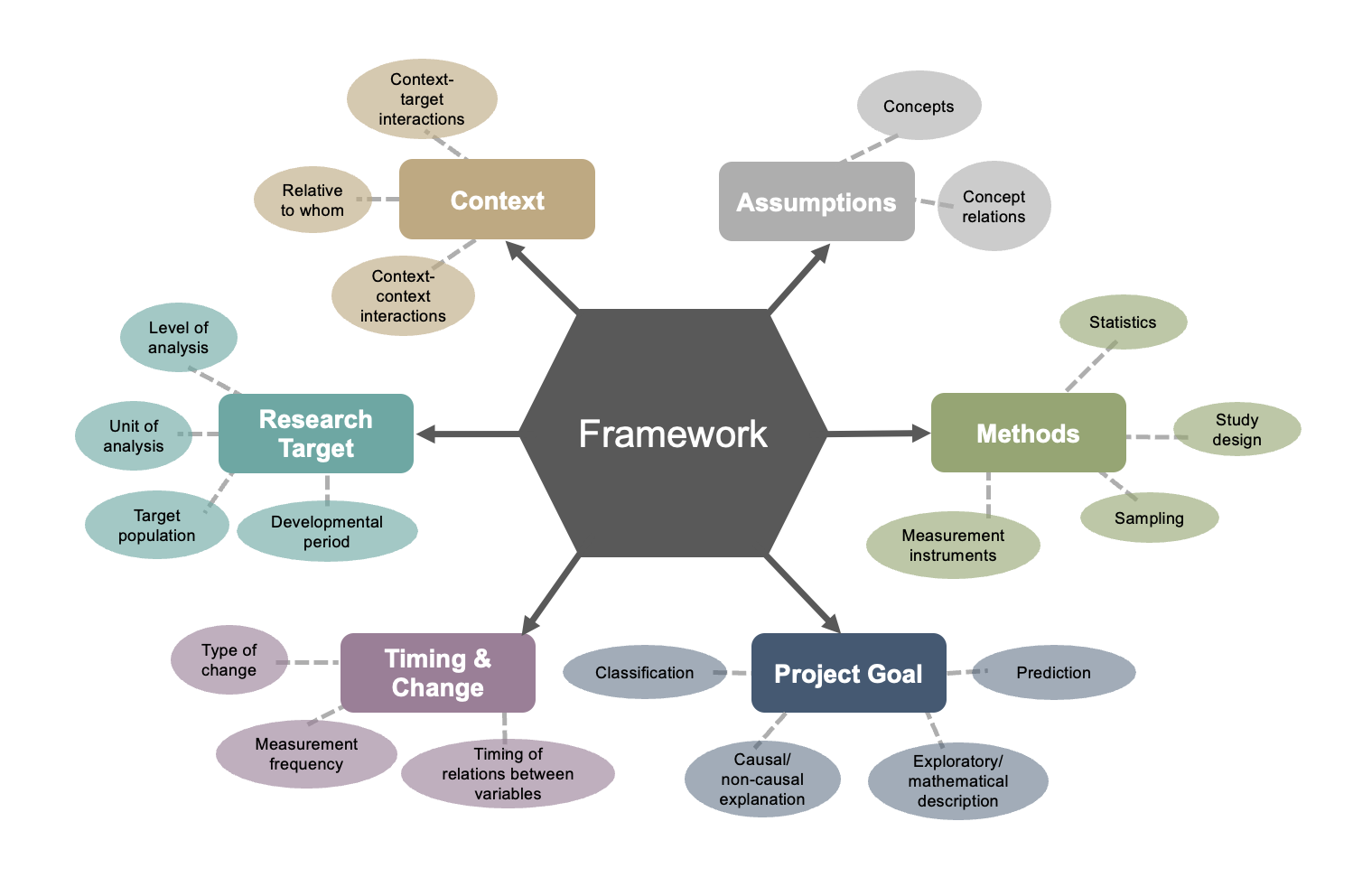


Write down your research project idea.

Write down a focal framework.

First, write down the most important ideas in the framework that you want to make sure to incorporate.

To connect the framework more explicitly to your project, write down the ideas from the framework that fall into each of the components of your project.

**Assumptions**

How does the framework inform the assumptions that will underlie your project? Consider the underlying “core” ideas of the framework –these can be high-level concepts about what exists or relations between concepts that are often not directly testable.

**Research Target**

How does the framework help you decide on the specific developmental topic or question that you wish to explore or explain (Lundberg et al., 2021)? Consider the units of analysis (e.g., parent vs child vs dyad), levels of analysis (e.g., behavior, brain, cognition, physiology, or relations between them), target populations (e.g., typically developing, autism spectrum), and/or developmental periods (e.g., infancy).

**Research Goal**

Does the framework help you decide the goal of your project? Consider whether you are after a causal or non-causal explanation, exploratory/mathematical description, predictive analysis, or classification of your developmental target (Ross, 2025).

**Context**

Does the framework help you think about what contexts are most important? Consider different contexts –ranging from the proximal (e.g., family) to distal (e.g., societal), who they are nested around, and which interactions among context(s) and research target are focal (e.g., mediation, moderation, constraints).

**Timing/Change**

Does the framework help guide which timescales are important for your project? Consider how frequently measurements are taken from your sample, what kind of change you are interested in, and the timescales at which variables relate to one another.

**Methods**

Does the framework guide your decision-making surrounding methods and how you implement them? Consider the study design, statistical models and their parameters, statistical estimand, and/or measurement instruments for studying your research target.

References and Resources

Lundberg, I., Johnson, R., & Stewart, B. M. (2021). What Is Your Estimand? Defining the Target Quantity Connects Statistical Evidence to Theory. *American Sociological Review*, *86*(3), 532–565.<https://doi.org/10.1177/00031224211004187>

Ross, L. N. (2025). Explanation in Biology. *Elements in the Philosophy of Biology*.<https://doi.org/10.1017/9781009300940>
